# Identification of TRP-Related Subtypes, Development of a Prognostic Model, and Characterization of Tumor Microenvironment Infiltration in Lung Adenocarcinoma

**DOI:** 10.3389/fmolb.2022.861380

**Published:** 2022-05-10

**Authors:** Sibo Sun, Yu Wang, Min Li, Jianqing Wu

**Affiliations:** Department of Geriatrics, The First Affiliated Hospital of Nanjing Medical University, Nanjing, China

**Keywords:** TRP superfamily, lung adenocarcinoma, tumor-immune microenvironment, overall survival, immunotherapy

## Abstract

The TRP (transient receptor potential) superfamily, as cation channels, is a critical chemosensor for potentially harmful irritants. Their activation is closely related not only to tumor progression and prognosis but also to tumor therapy response. Nevertheless, the TRP-related immune gene (TRIG) expression of the tumor microenvironment (TME) and the associations with prognosis remain unclear. First, we represented the transcriptional and genetic variations in TRIGs in 535 lung adenocarcinoma (LUAD) samples as well as their expression patterns. LUAD samples were divided into two distinct subtypes based on the TRIG variations. Significant differences had been found in prognosis, clinical features, and TME cell-infiltration features between the two subtypes of patients. Second, we framed a TRIG score for predicting overall survival (OS) and validated the predictive capability of the TRIG score in LUAD patients. Accordingly, to enhance the clinical applicability of TRIG score, we developed a considerable nomogram. A low TRIG score, characterized by increased immunity activation, indicated favorable advantages of OS compared with a high TRIG score. Furthermore, the TRIG score was found to have a significant connection with the TME cell-infiltration and immune checkpoint expressions. Our analysis of TRIGs in LUAD showed their potential roles in prognosis, clinical features, and tumor-immune microenvironments. These results may advance our knowledge of TRP genes in LUAD and show a new light on prognosis estimation and the improvement of immunotherapy strategies.

## Introduction

The TRP (transient receptor potential) superfamily of cation channels, at the very beginning, plays a crucial role in sensory physiology (Venkatachalam and Montell 2007). Mainly regulated by temperature, osmotic pressure, pH values, mechanical force, some endogenous and exogenous ligands, and intracellular signal molecules (Vay et al., 2012), TRP channels have been found to be expressed and functioned in smooth muscle cells of the bronchi, the pulmonary epithelium, the vasculature, and pulmonary endothelial cells (Taylor-Clark 2016). Nowadays, TRP channels receive potentially harmful irritants in addition to receiving sensory stimulation (Steinritz et al., 2018). When exposed to toxic matters, chemosensory TRP channels, as an important chemosensor for potentially harmful irritative substances, participate in cellular defense mechanisms, thereby affecting cell survival by regulating apoptosis. Toxic inhaled substances involved in lung cancer, such as acrolein, nicotine, nitric oxide, and other components in cigarette smoke, are recognized as TRP channel activators. They further activate *Akt* and *MAPK* signaling pathways through TRP channels (Buch et al., 2018). Also, increased cellular resistance to oxidative stress in lung cancer spheroids is linked to the high expression of *TRPA1*, which is a member of the TRP family (Takahashi et al., 2018).

As the primary subtype of lung cancer, non-small-cell lung cancer (NSCLC) constitutes over 80% of all lung cancers, with lung adenocarcinoma (LUAD) as its primary histological subtype. Despite clinical applications of targeted therapy and immunotherapy, the 5-year overall survival (OS) of LUAD patients remains at 16% as usual (Wood et al., 2016; Bray et al., 2018). Importantly, immunotherapies, whose responses are often durable and come with light toxicity in most people, are now given importance in cancer. However, the responses from people with similar tumors can vary considerably (Zou et al., 2016). Therefore, developing specific prognostic methods for LUAD patients is vital in finding new therapeutic targets so as to improve survival and quality of life. The TRP channel, *TRPV3*, is reported to be overexpressed on NSCLC tissues, compared with para-carcinoma lung tissues. In addition, the overexpression of *TRPV3* is associated with worse survival (Li et al., 2016), and its Ca2+ signaling is important to T-cell activation and differentiation (Majhi et al., 2015). However, there is a lack of studies regarding the impact of the TRP family on immunity and the prognostic potential for LUAD patients.

In this study, we obtained a comprehensive intratumoral immune landscape by fully assessing the expression of TRP channels. First, 21 TRP genes were extracted by gene differential expression analysis between LUAD and normal lung tissues. A total of 535 LUAD patients were divided into two subtypes according to these differentially expressed TRP genes. Second, patients were then stratified into two risk groups according to differentially expressed genes (DEGs) and differentially expressed immunity genes (DEIGs) based on the two TRP-related subtypes. Finally, a nomogram was set up to characterize the immune infiltration and predict OS of LUAD, which might prognose patient responses to immunotherapy and outcomes.

## Materials and Methods

### Datasets

The RNA sequencing (RNA-seq) data of 59 normal human lung samples, along with 535 LUAD patients and their clinical information, were obtained from The Cancer Genome Atlas (TCGA) database, https://portal.gdc.cancer.gov/. We obtained the validation cohort RNA-seq data and clinical features from the Gene Expression Omnibus (GEO) database, https://www.ncbi.nlm.nih.gov/geo/ (ID: GSE3141, GSE31210, GSE30219, GSE37745). Patients lacking survival information were eliminated for further analysis.

### Identification of Differentially Expressed TRP Genes

A total of 28 TRP genes were selected from prior reviews (Venkatachalam and Montell 2007). Before comparison, we normalized the TCGA data to fragment per kilobase million (FPKM) values and identified 21 differentially expressed TRP genes by the “limma” package with a *p* value <0.05. The “maftools” package was used to show the mutation landscape. A PPI (Protein–protein Interaction) network of the 21 TRP genes was formed into Search Tool for the Retrieval of Interacting Genes (STRING), version 11.0. https://string-db.org/ [Accessed 30 July, 2021].

### Development of the TRP-Related Gene Molecular Subtypes

Consensus unsupervised subtyping analysis was applied to sort the LUAD samples out into two distinct subtypes by R package “ConsensusClusterPlus” based on TRP gene expression. To identify the clinical value of the two subtypes, we used the Kaplan–Meier analysis to draw the survival curve by “survival” and “survminer” R packages. The Log-rank test was applied to compare the difference between the survival curves. To investigate the differences in the two TRP-related gene subtypes in biological processes, gene set variation analysis (GSVA) was performed with the hallmark gene set (c2. cp.kegg.v7.2) derived from the MSigDB database. We assess the immune, stromal, and estimate scores and the fractions of 22 human immune cell subsets of LUAD patients by the Estimation of STromal and Immune cells in MAlignant Tumour tissues using Expression data (ESTIMATE) and the CIBERSORT algorithm. In addition, the ingle-sample gene set enrichment analysis (ssGSEA) algorithm was used to determine the levels of immune cell infiltration in the tumor microenvironment (TME).

### Development and Validation of the Prognostic TRIG Score

A total of 2,483 immune genes were obtained from the ImmPort Resorce website, https://www.immport.org/shared/genelists. DEGs and DEIGs between the two TRP-related gene molecular subtypes were obtained by the R “limma” package (|log2FC| ≥ 1 and FDR < 0.05). The TRP-related immune genes (TRIGs) between DEGs and DEIGs based on TRP-related subtypes assessed by the R “Venn” package were applied to estimate the prognostic features. The least absolute shrinkage and selection operator (LASSO)-Cox regression analysis was used to consider the kernel prognostic TRIGs by the R “glmnet” package. Also, the penalty parameter (λ) value was filtered by the lowest partial likelihood deviance with 10-fold cross-validation. The TRIG score for patients was calculated by the following formula:

Risk score = (−0.00384*CCL17 expression) + (−0.20651*CD40LG expression) + (−0.09076*CIITA expression) + (0.095940*STC1 expression) + (−0.01538*SCGB3A1 expression) + (−0.13689* GDF10 expression).

We divided the TCGA LUAD samples into two groups via the median risk scores and compared OS time through Kaplan–Meier analysis. Principal component analysis (PCA) and the t-distributed stochastic neighbor embedding (t-SNE) algorithm were assessed respectively by the “prcomp” function in the “stats” and “Rtsne” R package. The “time-ROC”, “survival”, and “survminer” R packages were applied to perform the time-dependent receiver operating characteristic (ROC) curve analysis.

To validate this prognostic TRIG score, we employed four LUAD GEO cohorts (GSE31210, GSE3141, GSE30219, and GSE37745). TRIG expressions were normalized by the “scale” function. Then, we calculated TRIG scores through the exact formula before. In these four GEO datasets, samples were separated into two risk groups for comparison to validate the TRIG score.

### Clinical Associations and Stratification Analyses of the TRP-Related Prognostic Model

Univariate and multivariable Cox regression models are used to assess the distinctive character of the TRIG score and clinical features (age, gender, and TNM stage). Moreover, we used the stratified analysis to identify whether the TRIG score was able to maintain the predictive capability in distinct groups (age, gender, and TNM stage).

According to these TRIGs, we estimated the content of tumor-infiltrating immune cells (TIICs) in TME and explored the correlations between the six genes in the TRIG score and portions of 22 TIICs as well as the differential immune checkpoint expression levels between low- and high-risk groups by boxplots.

### Foundation and Validation of a Nomogram

A predictive nomogram was formed by extracting clinical features along with TRIG score in accordance with the results of the independent prognosis analysis, and then, time-dependent ROC curves for 1-, 3-, and 5-year survivals were applied to estimate the nomogram. Moreover, we also used calibration plots to show the prognostic value between the 1-, 3-, and 5-year survivals predicted by the nomogram and clinical results.

### Statistical Analyses

We used the Mann–Whitney test to compare the immune cell infiltration and immune checkpoints expression between the two groups. R version 4.1.0. Statistical significance was set at *p* < 0.05 in all statistical analyses.

## Results

### Expression Variations and Genetic Changes of TRP Genes in LUAD

**FIGURE 1:**
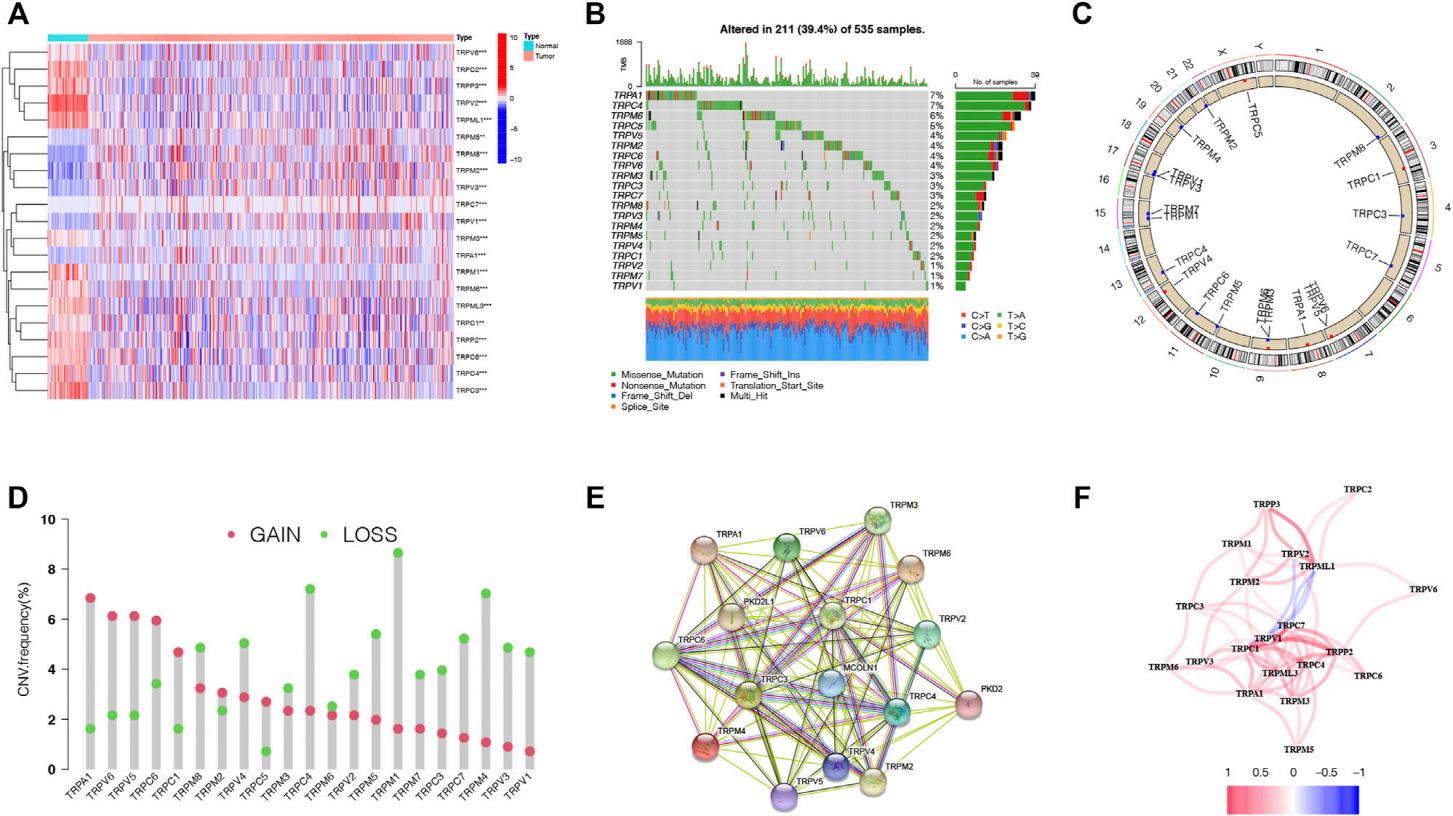
Expressions and correlations of 28 TRP genes in LUAD. **(A)**. Heatmap (red: high expression; blue: low expression) of the TRP genes between normal lung tissues (N, blue) and LUAD tissues (T, red). *p* values were demonstrated as **p < 0.01; ***p < 0.001. **(B)**. Mutation frequencies of TRP genes in LUAD patients from the TCGA cohort. **(C)**. Locations of CNV variations in TRP genes on 23 chromosomes. **(D)**. Frequencies of CNV, non-CNV and gain and loss CNV, among TRP genes. **(E).** PPI network demonstrating the interactions of the TRP genes (interaction score = 0.9). **(F)**. TRP gene correlation network (red string: positive correlation; blue string: negative correlation. The shades of the colors represent the correlation strength).

The expression levels of the 28 TRP genes (TRPs) were compared with The Cancer Genome Atlas (TCGA) data from 59 normal lung tissues and 535 tumor tissues, and 21 differentially expressed TRP genes were identified (*p* < 0.01). The RNA expressions of these differentially expressed TRP genes were shown with heatmaps (Figure 1A, red: high expression; blue: low expression). Meanwhile, 21 TRP gene mutations were presented in 211 of the 535 samples (about 39.4%) at the genetic

expression level. *TRPA1* and *TRPC4* displayed the highest mutation frequency (Figure 1B). Copy number variations (CNVs) of all the 21 TRP genes were detected, and most TRP genes were gathered on copy number amplification (Figure 1C). Alterations of the 21 TRP genes with CNVs on the chromosome were also identified (Figure 1D). To explore the interactions of 21 TRP genes, a protein–protein interaction (PPI) analysis was conducted (Figure 1E). The value of 0.9 (the highest confidence) was set as the minimum required interaction score of the PPI analysis. *TRPC1*, *TRPC3*, *TRPC4*, *TPRC6*, *TRPM2*, and *TRPM3* were considered as hub genes. The correlation network of the 21 TRP genes was also detected (Figure 1F, red: positive correlations; blue: negative correlations). The results indicated that CNV changes might lead to abnormal gene expression. Also, the expression levels of TRP genes were linked with LUAD, suggesting that they might consider different characteristics in patients.

### Identification of Two Subtypes of LUAD Based on the 21 TRP Genes

**FIGURE 2:**
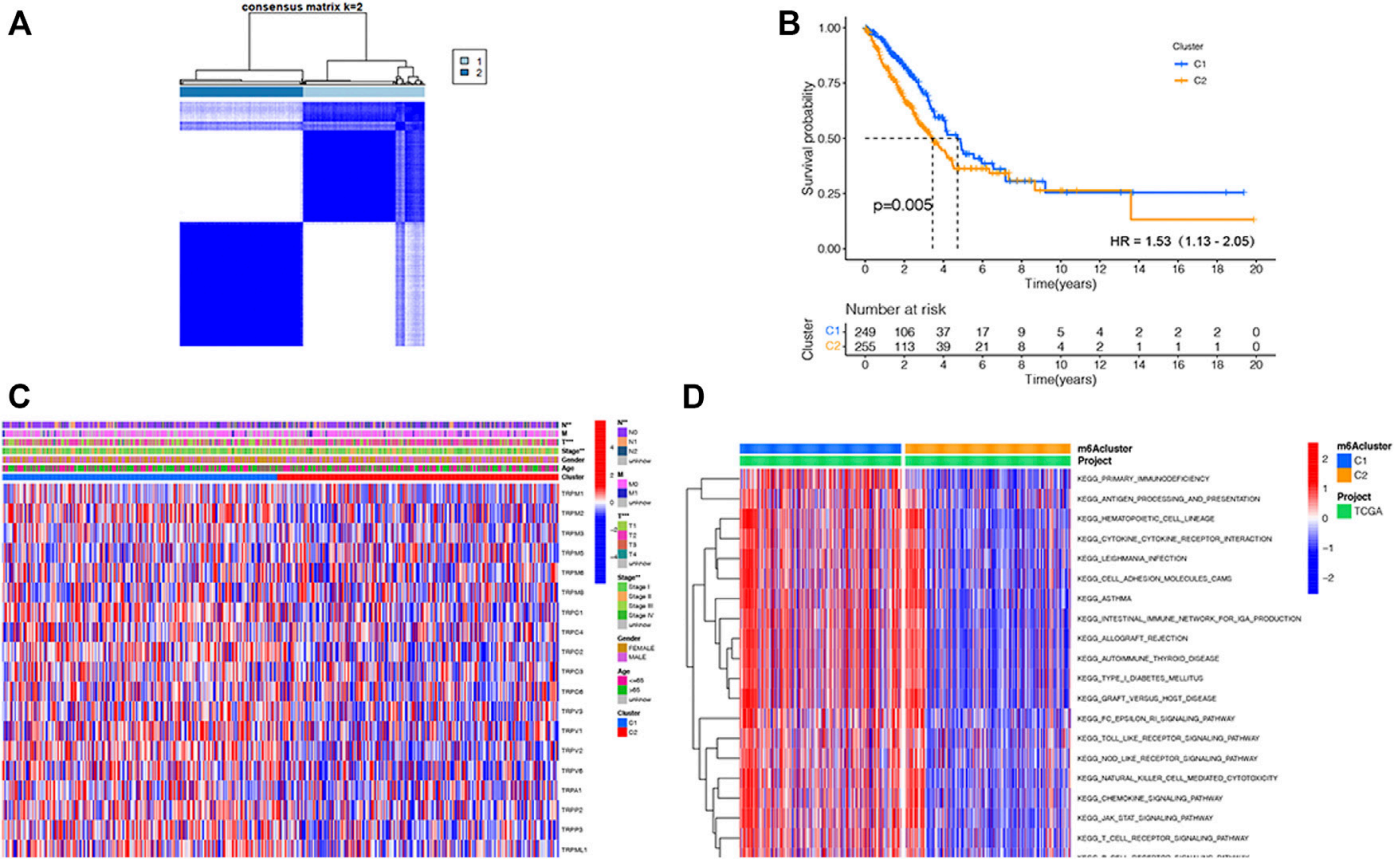
TRP-related subtypes and their clinicopathological features and biological pathways. **(A)**. Consensus matrix heatmap identifying two subtypes (*κ* = 2). **(B)**. Kaplan–Meier OS curves of the two subtypes. **(C)**. Variations in clinicopathologic characteristics and TRP gene expression levels between the two distinct subtypes. **(D)**. Biological pathways to two distinct subtypes *via* GSVA (red: activated pathways; blue: inhibited pathways).

Considering the important functions of the TRP family, we conducted the consensus clustering of the 535 LUAD samples based on the TRP family to explore new biological functions. We increased the clustering variable (κ) from 2 to 9 and found that when κ = 2, the intergroup correlations were the lowest and intragroup correlations were the highest, indicating that the 535 LUAD patients could be compartmentalized into two subtypes according to the 21 TRP genes (Figure 2A). Between the two subtypes, most TRP gene expression levels were higher in subtype 1 (Supplementary Figure S1). Meanwhile, survival benefit of subtype 1 was higher than that of subtype 2 (HR = 1.53, 95% CI: 1.32–2.05. Figure 2B). We presented a heatmap of the gene expression profile, along with the clinical features. High expression levels of most TRP genes were identified in subtype 1. Also, subtype 1 was found to have a lower degree of tumor invasion, lymph node metastasis, and distant metastasis. Also, clearly, subtype 2 represented a later stage compared with those in subtype 1. Furthermore, most TRP gene expression levels were higher in subtype 1 (Figure 2C). We performed GSVA enrichment analysis to consider the variations in biological behavior between these two subtypes (Figure 2D). Subtype 1, compared with subtype 2, demonstrated the enrichment in respect of pathways linked with activation of the immune system (Barclay 2003; Lord et al., 2003; Olivier et al., 2005; Akdis et al., 2016). The results revealed that the two subtypes could be distinguished by the 21 TRP genes, and the lower survival advantage of subtype 2 is mainly related to disorders of the immune system.

### TME Infiltration of the Two TRP-Related Subtypes

**FIGURE 3:**
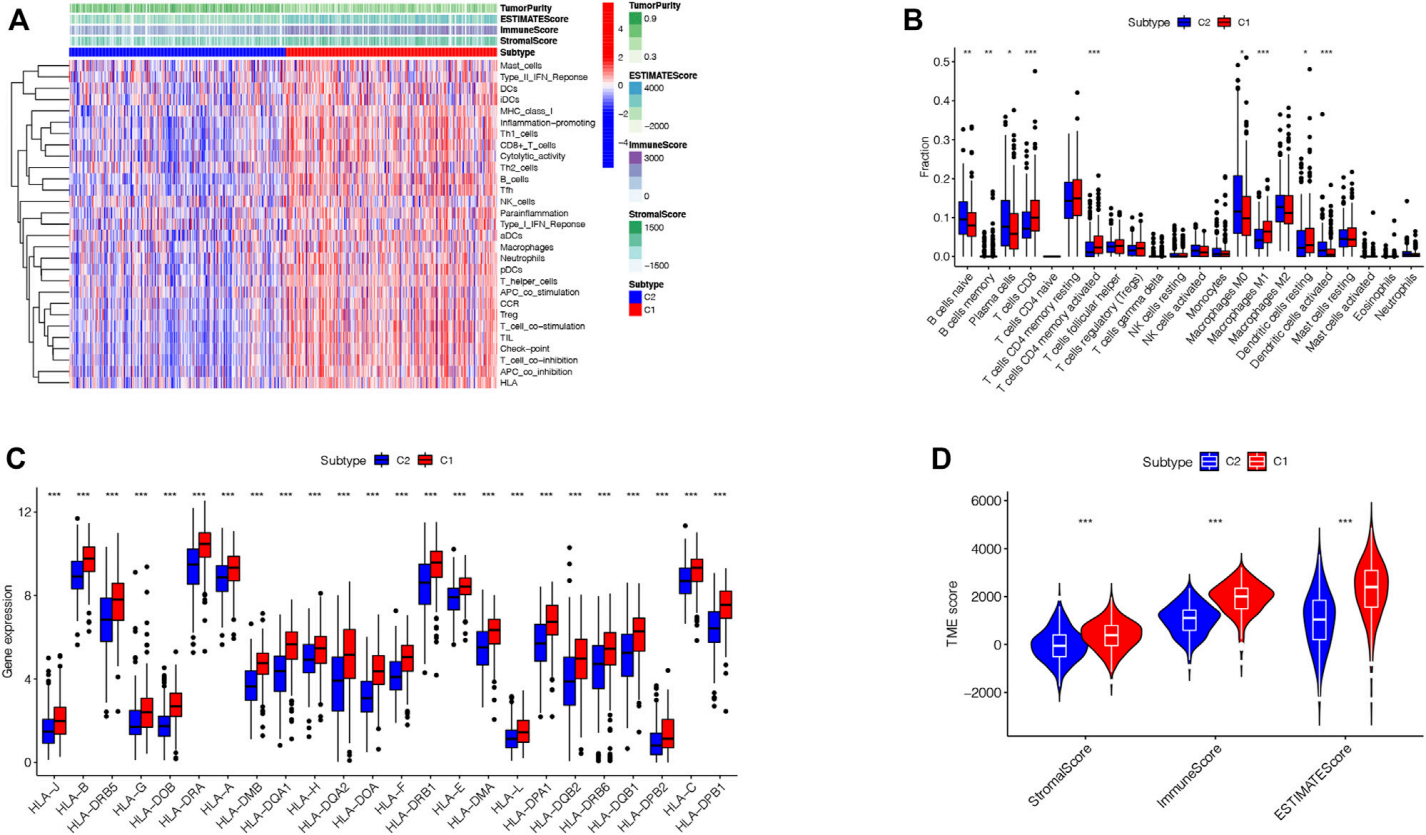
TME infiltration in two subtypes. **(A).** Immunological characteristics of the two subtypes. **(B)**. Correlations of the immune cell infiltration level between two subtypes. **(C)**. Correlations of HLA gene expression level between two subtypes (*p < 0.05, **p < 0.01, ***p < 0.001). **(D)**. Correlations of Stromal-score, Immune-score, ESTIMATE-Score, and TME score between two subtypes.

To explore the immunological features of the two subtypes, 535 LUAD samples of the TCGA cohort were analyzed by ssGSEA analysis with 29 immune gene sets. The TME is considered as the complex multicellular environment in tumor development. It comprises immune cells, including T- and B-lymphocytes, tumor-associated macrophages (TAMs), dendritic cells (DCs), natural killer (NK) cells, neutrophils, myeloid-derived suppressor cells (MDSCs), stromal cells, the extracellular matrix (ECM) and other secreted molecules, and the blood and lymphatic vascular networks (Junttila and de Sauvage 2013). Between them, the immune cells in the TME play vital roles in possessing tumor-antagonizing or tumor-promoting functions (Quail and Joyce 2013).TME features of these two subtypes were identified using the ESTIMATE algorithm. The outcomes demonstrated that subtype 1 had higher expressions of all TME scores, while subtype 2 had lower expressions of these scores (Wilcox test, *p* < 0.001) (Figure 3A). Next, we explored immune cell infiltrations by implementing the CIBERSORT algorithm. Subtype 1 showed the enrichment of the activated innate immune cell infiltration, comprising the presence of CD8 T and activated CD4 cells, M1 macrophages, memory B-cells, and resting dendritic cells, thus meeting a significant survival benefit. Subtype 2 was abundant with naive B-cells, plasma cells, M0 macrophages, and activated dendritic cells (Figure 3B). We drew to the conclusion that these two subtypes had entirely different human leukocyte antigen (HLA) infiltration characteristics. HLAs are highly polymorphic alloantigens that encode the product of a gene cluster encoding the human major histocompatibility complex (MHC) (D.S. Chen and Mellman 2017). Neoantigens produced by tumors must first be presented on HLAs and recognized by peptide-specific receptors. Then, HLAs and the peptide-specific receptors form the MHC-antigen peptide-specific receptor complex, participating in regulating the immune response of the body (Kalaora et al., 2021). All the HLA gene expression levels were significantly higher in subtype 1 but lower in subtype 2 (Wilcox test, *p* < 0.05), indicating that subtype 1 was inclined to generate protective immunity (D.S. Chen and Mellman 2017) (Figure 3C). Besides, to presume the tumor purity of the two subtypes, TME scores (stromal score, immune score, and estimate score) of the two subtypes were investigated through the ESTIMATE package. For TME scores, the higher stromal scores or immune scores, the higher contents of stromal cells or immunocytes in the TME, and estimated scores represented the comprehensive scores of stromal or immune scores in the TME (Han et al., 2022; W.; Chen et al., 2021) (Figure 3D). Our outcomes proved that subtype 1 possessed higher TME scores.

### Development of a Prognostic TRIG Score in the TCGA Cohort

**FIGURE 4:**
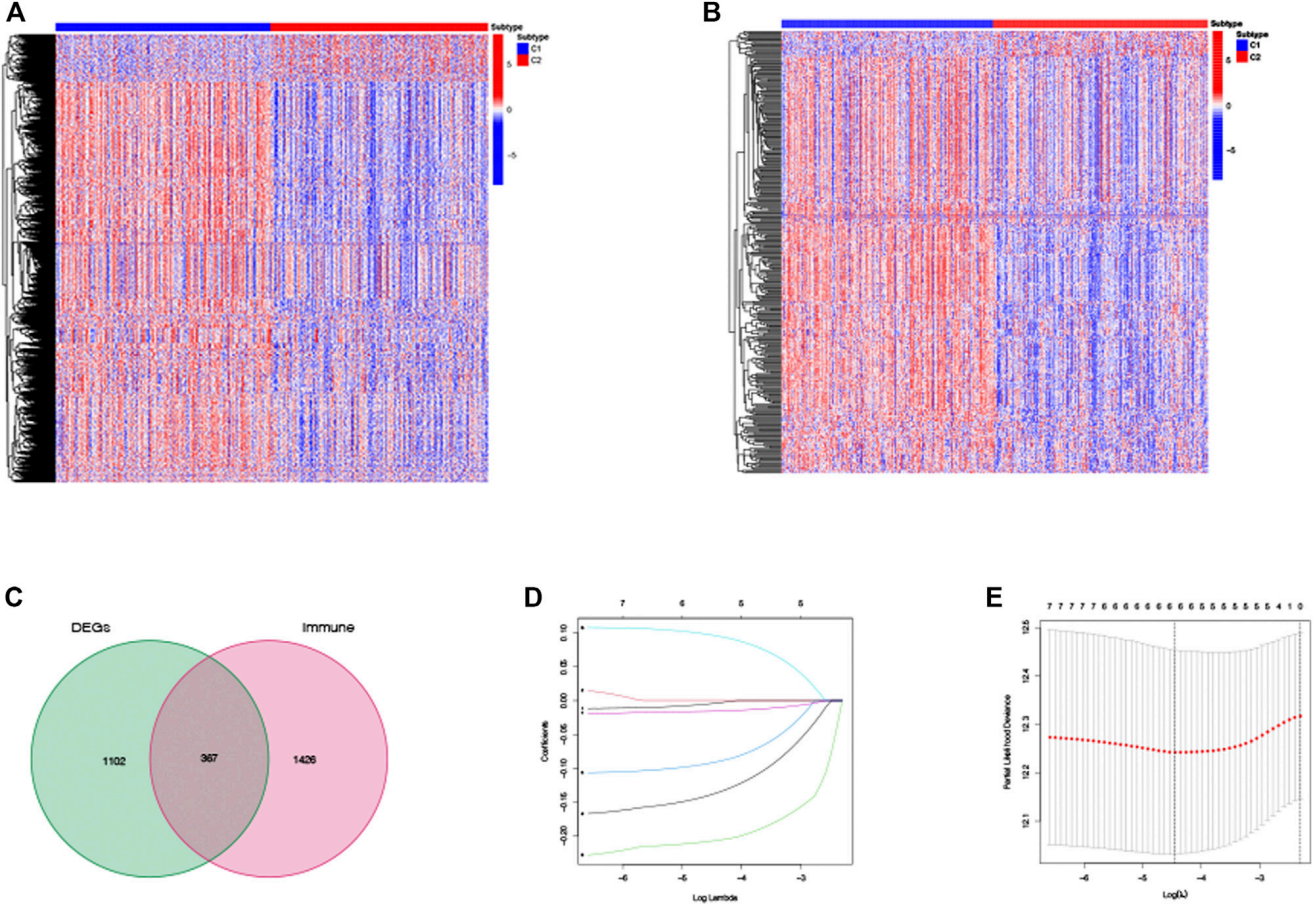
DEG and DEIG expressions and establishment of the prognostic TRIG score in the TCGA cohort. **(A,B)**. Heatmaps of DEGs and DEIGs between two subtypes. **(C)**. TRIGs between two subtypes. **(D)**. LASSO regression of the six TRIGs. **(E)**. Cross-validation of parameter selection of the LASSO regression.

**FIGURE 5:**
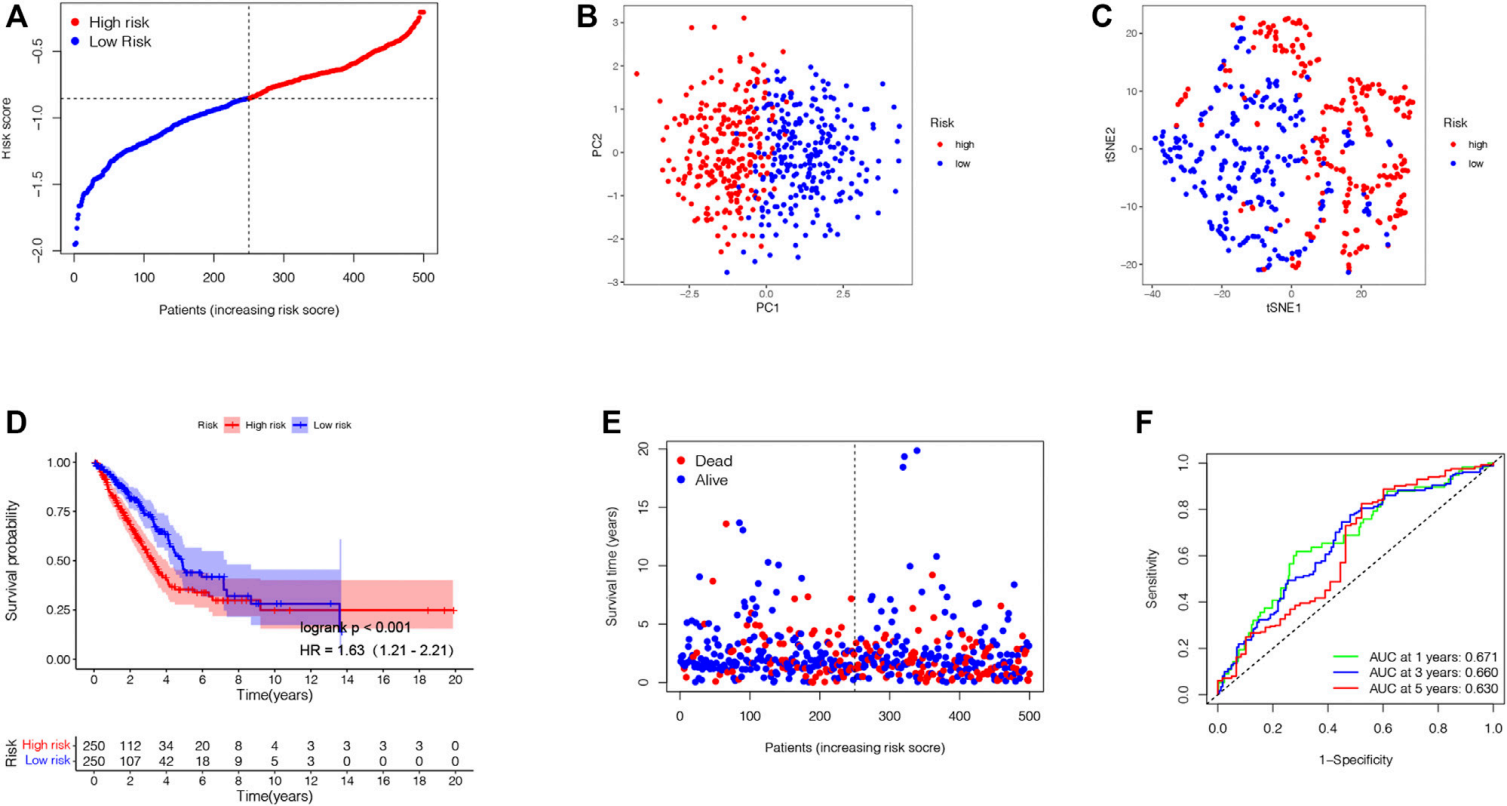
Establishment of the prognostic TRIG score in the TCGA cohort. **(A)**. Spread of survival status and risk scores of the TCGA cohort. **(B,C)**. Variations in the distribution of different TRIGs risk groups. **(D)**. Kaplan–Meier survival curve of OS of LUAD samples in the high- and low-risk groups. **(E)**. Survival status for each patient in low-risk and high-risk populations. **(F)**. AUC of time-dependent ROC curves showed the predictive efficiency of the TRIG score.

Based on those two TRP-related subtypes, we explored the features of tumor-immune interactions and their prognostic potential for LUAD samples. First, 1,469 DEGs were identified with the two subtypes (Figure 4A). Subsequently, 1,793 genes were considered as DEIGs according to the ImmPort database (Figure 4B). The 367 intersect genes between DEGs and DEIGs were applied to estimate the prognostic features (Figure 4C). In order to build a prognostic TRIG score to evaluate each patient, we extracted six of the 367 TRP-related genes by utilization of the LASSO-Cox regression model along with a minimum of λ (Figures 4D,E). The formula was identified as follows: risk score = (−0.00384*CCL17 expression) + (−0.20651*CD40LG expression) + (−0.09076*CIITA expression) + (0.095940*STC1 expression) + (−0.01538*SCGB3A1 expression) + (−0.13689* *GDF10* expression). We used the formula to calculate the median score, according to which two risk groups were then separated from 535 patients (Figure 5A). Dimensionality reduction algorithms of PCA (Figure 5B) and t-SNE (Figure 5C) were used to show discernible dimensions between the low- and high-TRIG-score groups. PCA and t-SNE analysis revealed significant differences between the two subtypes. We performed survival analysis for two risk groups by Kaplan–Meier curves. The high-risk group showed a poorer survival time than the low-risk group (HR = 1.63, 95% CI: 1.21–2.21. Figure 5D). We identified a significant variation in survival times and survival statuses of the two groups by the Kaplan–Meier curves. The distribution plot of the risk of TRIG score demonstrated that the survival times decreased, while mortality increased with an increased TRIG score (*p* < 0.001, Figure 5E). In addition, we used time-dependent ROC analysis to estimate the predictive efficacy of the TRIG score. The area under the ROC curve (AUC) reached 0.671 for 1-year survival, 0.660 for 2-year survival, and 0.630 for 3-year survival (Figure 5F).

### Validation of the Prognostic TRIG Score in the GEO Cohorts

**FIGURE 6:**
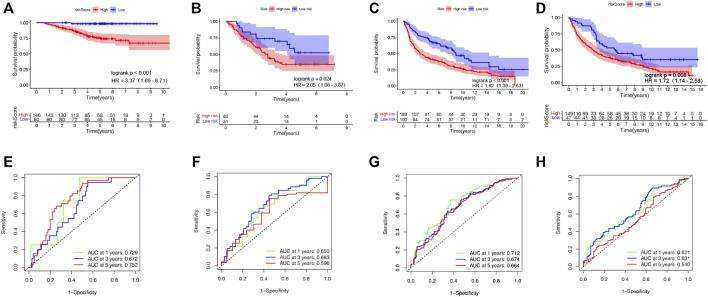
Validation of the prognostic TRIG score in various GEO cohorts of LUAD. **(A-D).** Kaplan–Meier survival curves of OS in four GEO cohorts. **(E-H).** Time-dependent ROC curves for four GEO cohorts. **(A,E)**. GSE31210 (*n* = 226). **(B,F)**. GSE3141 (*n* = 111). **(C,G).** GSE30219 (*n* = 289). **(D,H)**. GSE37745 (*n* = 196).

Aiming to confirm the reproducibility and stability of prognostic TRIGs of LUAD, we derived the TRIG expression levels and LUAD samples’ clinical record from four independent LUAD cohorts from GEO databases (GSE3141, GSE31210, GSE30219, and GSE37745). We calculated the LUAD patients’ TRIG scores in the four GEO databases by the median risk score gained before. Kaplan–Meier survival analysis demonstrated that the OS in the low-risk group was significantly better than that of the high-risk group of the four GEO databases (HR = 3.37, 95% CI: 1.69–6.71. Figure 6A; HR = 2.05, 95% CI: 1.08–3.87. Figure 6B; HR = 1.92, 95% CI: 1.39–2.63. Figure 6C; HR = 1.72, 95% CI: 1,14–2.59. Figure 6D). Meanwhile, ROC curve analysis made clear that the prognostic TRIG score had good predictive efficacy (Figures 6E–H).

### Independent Prognostic Value of the Prognostic TRIG Score

**FIGURE 7:**
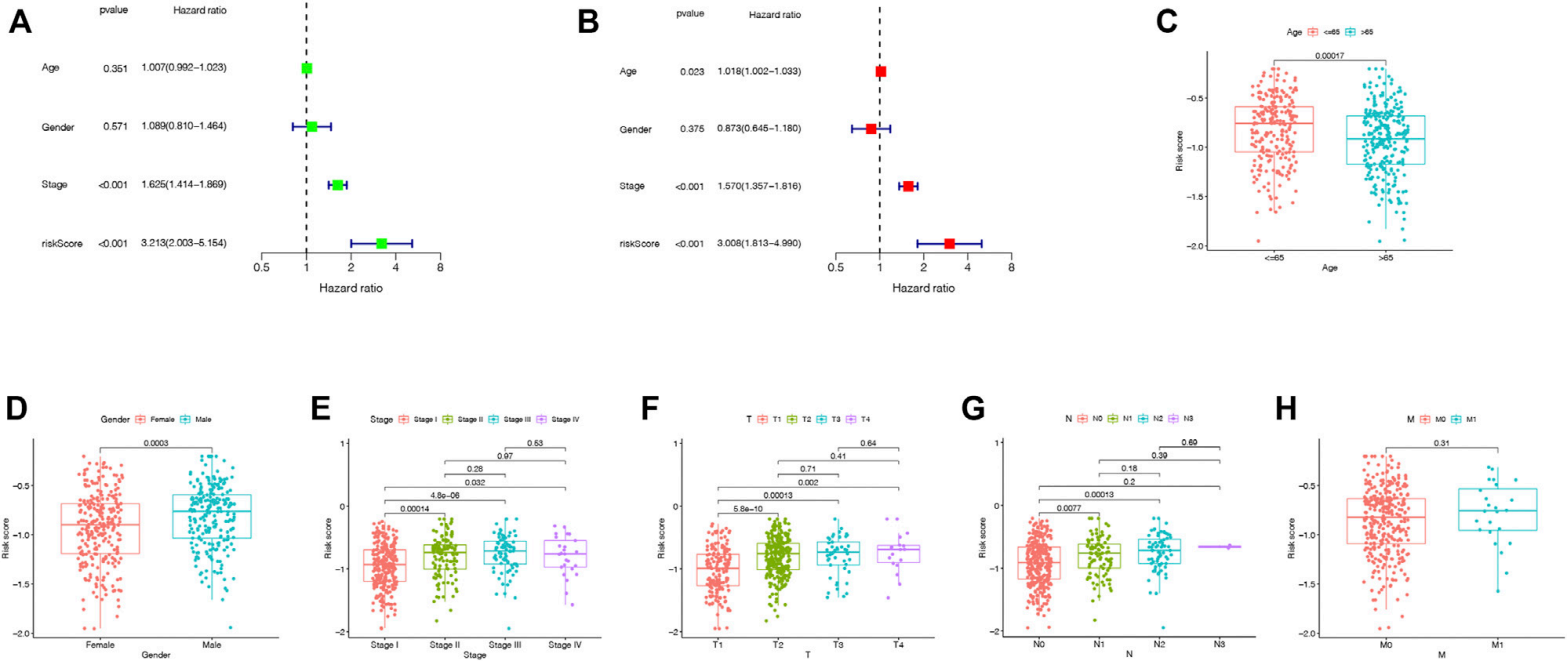
**(A,B)**. Univariate and multivariate Cox regression analysis of the prognostic TRIG score in the TCGA cohort. **(C-H)**. Connections between clinical features and the risk groups.

In the TCGA cohort, the univariate Cox regression analysis showed that the TRIG score was a prognostic factor for LUAD patients (HR = 3.213, 95% CI: 2.003–5.154. Figure 7A). Meanwhile, the multivariate analysis indicated that the TRIG score was an independent factor of survival prediction (HR: 3.008, 95% CI: 1.813–4.990. Figure 7B). We still estimated the relationship between TRIG score and clinical traits, including age, gender, and TNM stage in the TCGA cohort (Figures 7C–H). All these clinical features, except instance metastasis, were linked with TRIG score. The results indicated that advanced LUAD samples had a higher TRIG score than early LUAD samples. Together, these outcomes indicated that the TRIG score was positively associated with tumor stages, suggesting that TRIG score showed the potential as a clinical indicator to assess the LUAD patient survival rates.

### Interrelation of the TRIG Score and Immune Activity

**FIGURE 8:**
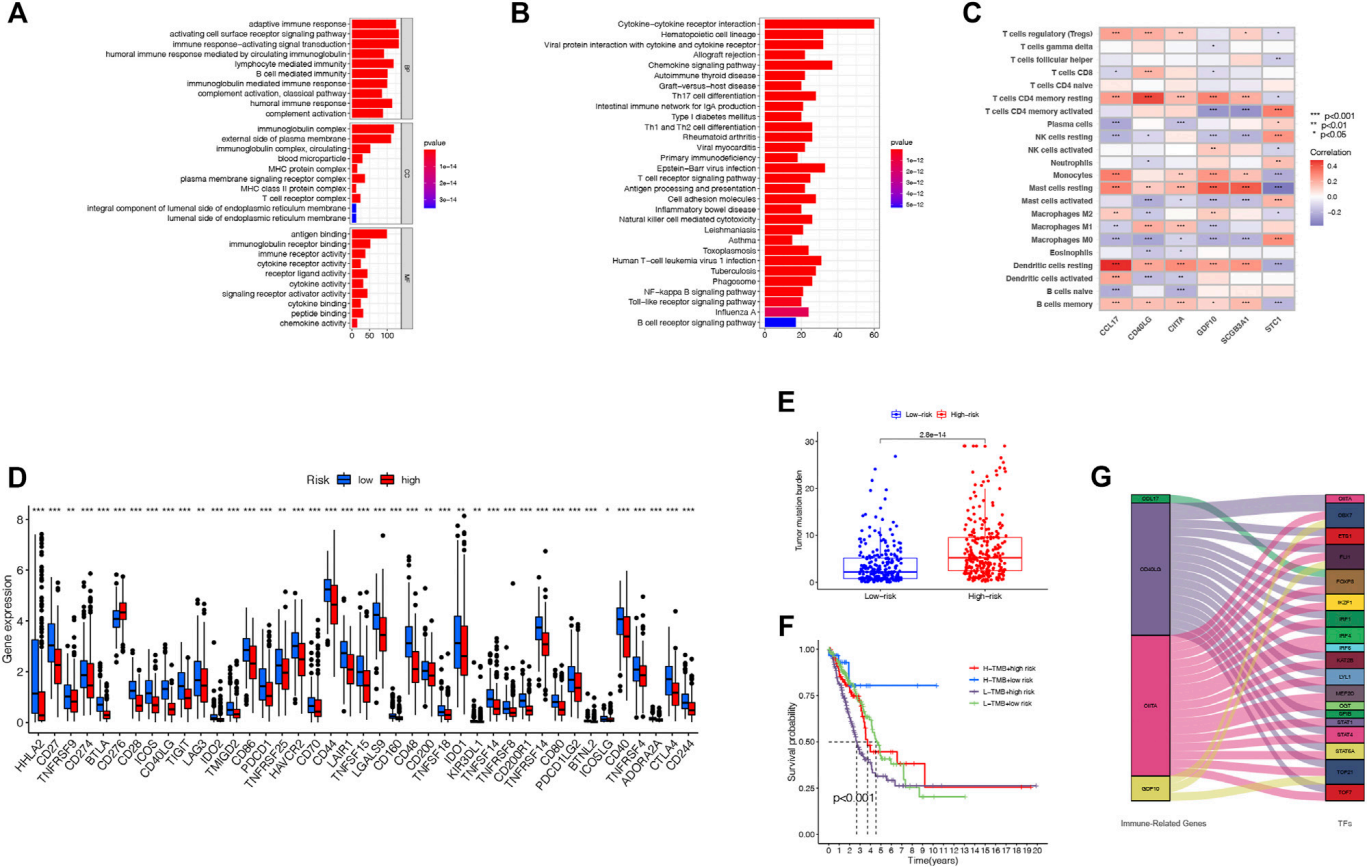
Functional analysis and evaluation of immune activity between the two groups. **(A)**. GO analysis of TRIGs between various risk groups. **(B)**. KEGG analysis of TRIGs between various risk groups. **(C)**. Relationship between TRIG score and immune cell types. **(D)**. Expression of immune checkpoints in the high- and low-risk groups. **(E)**. Comparison of TMB between high- and low-risk groups. **(F)**. Comparison of four groups stratified by combining the TMB and risk groups. **(G)**. Regulatory network of the TRIGs and TFs.

We investigate the variations in the gene functions as well as pathways between the two risk groups. The outcomes of Gene Ontology (GO) enrichment analysis and Kyoto Encyclopedia of Genes and Genomes (KEGG) pathway analysis based on 367 TRIGs demonstrated that TRIGs were mainly linked with the immune response, chemokine-mediated signaling pathways, and inflammatory cell chemotaxis (Figures 8A,B). Next, we explored the correlations between the TRIG score and immune cell infiltration, TMB (tumor mutational burden), tumor transcription factors (TFs), and immune checkpoints. On one hand, we explored the correlations between the prognostic TRIG score and the enrichment of TIICs by the Pearson correlation analysis, which referred to the infiltrating immune cells that could be isolated from the tumor tissue when immune cells moved from the blood to the tumor tissue. The infiltration of immune cells in tumors was closely related to clinical prognosis, and immune cells infiltrated in tumors were most likely to serve as immunotherapy targets (Domingues et al., 2016). We found that most TIICs were linked with the six genes (Figure 8C). On the other hand, we extracted immune checkpoint genes from prior reviews (Kraehenbuehl et al., 2022; He and Xu 2020; Pardoll 2012) to evaluate their relationships with the TRIG score with the purpose of investigating whether the TRIG score was able to predict the benefits of immune checkpoint inhibitors of LUAD patients via the CIBERSORT algorithm. The results demonstrated that besides *CD276*, all the immune checkpoint expressions were negatively linked with the TRIG score (Figure 8D), indicating that TME suppression might be linked with the poor prognosis of high-TRIG patients. TMB was defined as the total number of somatic gene coding errors, base substitutions, and gene insertion or deletion errors detected per megabase and is considered as a biomarker for evaluating the efficacy of PD-1 antibody therapy (Cristescu et al., 2018). Based on the Kruskal–Wallis rank-sum test, the “ggpubr” package in the R language was run to explore the relationship between TMB score and risk groups. We found that the high-risk group showed higher TMB, compared with the low-risk group (Figure 8E). We combined the TRIG score and TMB to improve the efficiency of predictive prognosis and stratified all the patients into high TMB/low-risk, high TMB/high-risk, and low TMB/high-risk groups. Significant differences were detected among four groups (Log-rank test, *p* < 0.001). TMB was considered to be a biomarker that could predict the immune checkpoint inhibitors’ efficacy, and immune checkpoint inhibitors were proved to be more efficient in the TMB-high subgroup in LUAD (Sha et al., 2020), consistent with our findings that patients in the low TMB/high-risk group showed the worst prognosis compared to the high TMB/high-risk group. There was no significant difference between high TMB/high risk and low TMB/low risk (Figure 8F). To explain the role of the immune molecule regulatory network in the process of LUAD, we assessed the relationships between LUAD development-related TFs and the six TRIGs. TFs related to tumorigenesis and the development of LUAD were obtained from the CISTROME project. Then, we extracted the differentially expressed TFs from the intersect genes between DEGs and DEIGs and used Pearson’s correlation coefficient analysis to construct the regulatory network of the TFs and the six TRIGs. ∣r∣ > 0.3 and FDR < 0.01 were set as the cutoffs for a significant correlation. Four of six TRIGs were linked with the corresponding 19 TFs (Figure 8G). Therefore, it is reasonable to conclude that immune cell infiltration was significantly linked with the TRIG scores, which might affect the LUAD patient prognosis.

### Advancement of a Nomogram to Predict Survival

**FIGURE 9:**
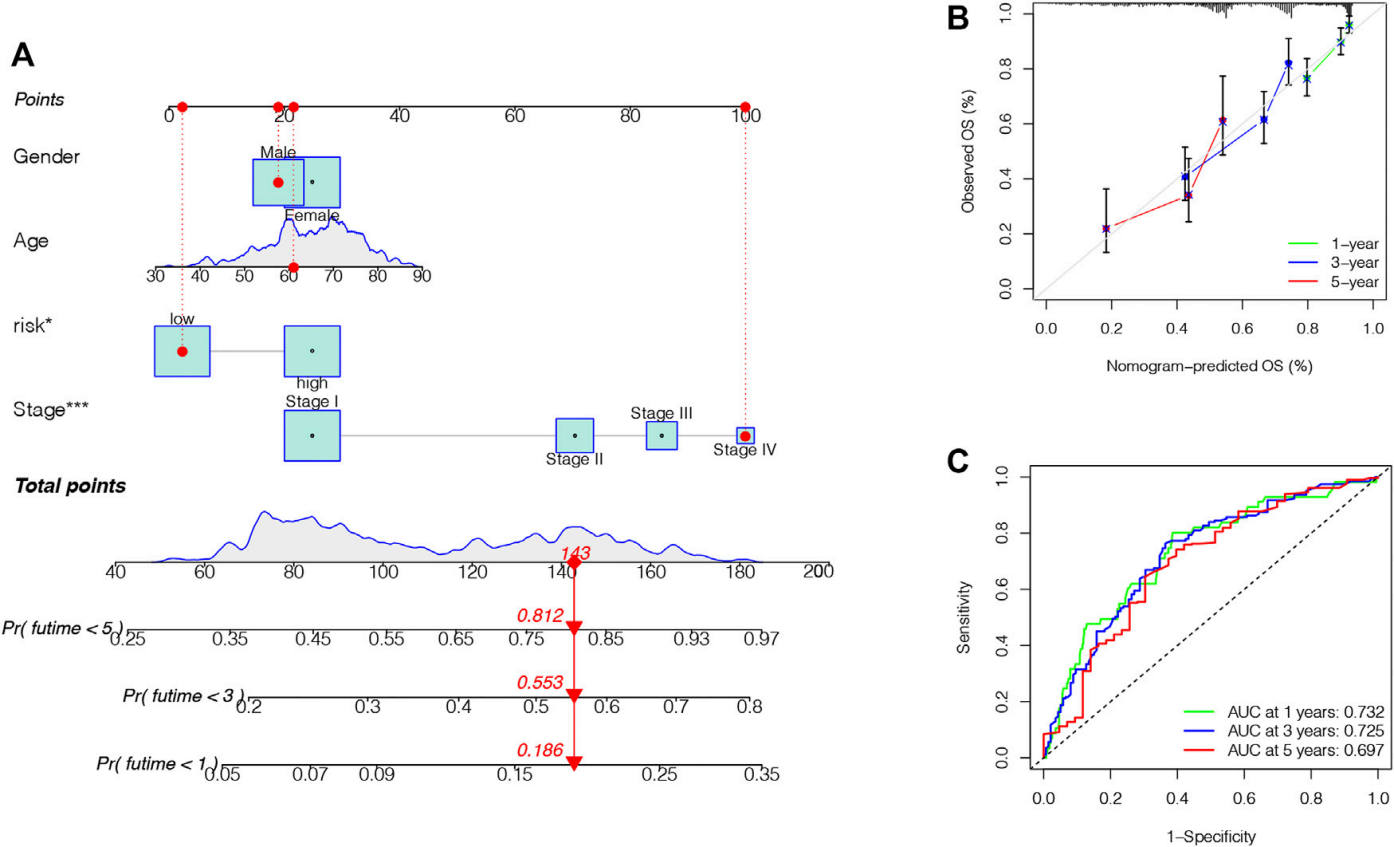
Construction and validation of a nomogram. **(A)**. Nomogram for predicting the 1-, 3-, and 5-year OS of LUAD patients. **(B)**. ROC curve and AUC of the predictions for 1-, 3-, and 5-year OS in the TCGA cohort. **(C)**. Calibration curves of the nomogram for the TCGA cohort.

Finally, we formed a nomogram to expand the scope of the TRIG score clinical application in predicting OS in LUAD patients (Figure 9A). According to the gender, age, risk (“low risk” represented “low TRIG score”; “high risk” represented “high TRIG score”), and stage, the total point values of every patient were calculated by prognostic parameters. With the increase of the patient total points, the clinical prognosis became worse. The ROC curve supported the good predictive value of the nomogram (Figure 9B). Moreover, the calibration plot indicated that the nomogram had a similar performance compared with an ideal model (Figure 9C).

## Discussion

Plenty of research studies have disclosed that the TRP channels played an indispensable role in anti-tumor immune effects (Santoni and Farfariello 2011; Parenti et al., 2016). Most of their major focuses, however, were on a single TRP gene. The total effect and immune infiltration features intervened by the multiple TRP genes have not been fully illustrated. Our study showed variations in TRP genes at the genetic and transcriptional levels in LUAD. According to the differently expressed TRP genes, we classified patients into two subtypes. Between them, subtype 2 patients showed more severe clinical features and worse survival rates. Furthermore, the differences in TME features between two distinct subtypes were significantly linked with immune-related biological pathways, and subtype 1 was identified by a significant immune activation. Therefore, our studies showed that TRP genes might assume the role of a predictor for estimating the immunotherapy response and clinical outcome of LUAD. We further classified LUAD samples in the TCGA into two risk groups based on the DEGs and DEIGs between the two TRP-related subtypes. Moreover, we formed the prognostic TRIG score and revealed its predictive capability. Next, we explored the six TRIG score gene expression levels in LUAD. Patients with low and high TRIG scores were represented, respectively, as immune stimulation and suppression. Moreover, they showed significant differences in prognosis and clinical characteristics, also in the expressions of TME, TMB, and immune checkpoints. Last, by combining the clinical features and TRIG score, we set up a nomogram to facilitate the clinical use of the TRIG score. The prognostic TRIG score will be able to promote a better understanding of the LUAD molecular mechanism as well as provide new inspirations for anti-cancer therapies.

TME is a compound of tumor cells and their ambient cells, primarily composed of TIICs, the tumor vasculature, ECM, lymphocytes, cancer-associated fibroblasts, and bone marrow-derived inflammatory cells. Tumor development, progression, and therapeutic resistance were also reported to be significantly impacted by TME (Quail and Joyce 2013). Immune cells, as significant cellular elements of TME, were engaged in multiple immune activities and responses. For instance, the tumor-related inflammation that can prevent tumor progression is regulated by the immune system (Ribeiro Franco et al., 2020). Cytotoxic T-cell activation in the TME is considered to play vital roles in possessing tumor-antagonizing or tumor-promoting functions (Pardoll 2012). B-cells have been reported to both suppress and support T-cell functions, leading to differential effects on tumorigenesis (Ammirante et al., 2010). Moreover, B-cells have also been shown to promote tumor progression by enhancing pro-tumoral inflammation (Nelson 2010). Mast cell recruitment is related to tumorigenesis and angiogenesis (Coussens et al., 1999; Mantovani and Sica 2010). TAMs can also affect tumor progression depending on their polarization (Yang et al., 2008). LUAD patients are response heterogeneity to immunotherapy; particularly, those with highly expressed tumor neoantigens, tumor-infiltrating lymphocytes, and checkpoints tend to have a poor prognosis (Rosenberg et al., 2011; Rizvi et al., 2015; Verdegaal et al., 2016; Berner et al., 2019; Arrieta et al., 2020), showing the essential role of TME in LUAD. The TRP channel, *TRPV1*, is proven to be a major Ca2+ channel. In addition, Ca2+ ions, which contributed the important inter-and intracellular messengers to the TME, have been investigated elsewhere (Bong and Monteith 2018; Roberts-Thomson et al., 2019). In this study, the TRP pattern identified by immune suppression showed a higher TRIG score, while, on the contrary, the pattern featured by immune activation was linked with a lower TRIG score. Hence, we discovered that TME features, including the relative abundance of 22 TIICs, varied significantly from two subtypes and different TRIG scores, indicating the key role of TRP genes in LUAD progression.

The tumor-infiltrating T-cell enrichment in LUAD tissues is higher compared with those in normal tissues, and higher enrichment of tumor-infiltrating T-cells indicates a good prognosis (Guo et al., 2018; Stankovic et al., 2018). CD4^+^ T cells are crucial to driving not only the antibody but also cytotoxic CD8^+^ T cell response. Moreover, they promote an inflammatory environment that favors antitumor immunity (Tran et al., 2014). Among them, memory CD4^+^ T cells are reported to play a crucial role in anti-tumor responses to LUAD (Dieu-Nosjean et al., 2016). In this study, the low-risk group and the low TRIG score, accompanied by a higher survival rate, revealed higher resting memory CD4^+^ T cell expression. At the first time, resting memory CD4^+^ T cells are extracted from activated T-cells, and then they encounter antigens, followed by multiplying to produce a stronger and faster immune response to, in the second response, the experienced antigens. It has been proved that resting memory CD4^+^ T cells can regulate tumor growth (McKinstry et al., 2010), which corresponds to our findings. Therefore, we assume that a high percentage of resting memory T-cells can strongly activate effector T-cells and thus favors an ideal result. In addition, the proportion of resting memory CD4^+^ T cells increased in LUAD patients younger than 65 years old and non-smoking. Nevertheless, resting memory CD4^+^ T cells can be partitioned into at least five subsets of cells, and which subtypes of memory CD4^+^ T cells are linked with LUAD prognosis is yet to be found.

B-cells are also proved to participate in the immune response. Evidence showed that enrichment of tumor-infiltrating B-cells is the most powerful prognostic factor of prolonged survival and is strongly linked to *PD-1* blockade responses in soft-tissue sarcomas (Petitprez et al., 2020). Furthermore, tumor-infiltrating B-cells are detected at low levels, accompanied by a poor prognosis in advanced NSCLC (J. Chen et al., 2020; Germain et al., 2014). Meanwhile, higher expressions of B-cell-related genes *IGLL5*, *MZB1*, and *JCHAIN* are identified in patients with responding immune checkpoint blockade than those in non-responders (Helmink et al., 2020). In this study, no significant difference in native B-cell infiltration was found between the two risk groups, while the enrichment of memory B-cells in the low-TRIG-score group with longer OS was significantly higher than those in the high-TRIG-score group. The generation of memory B-cells is reported as a key characteristic of the adaptive immune system. Memory B-cells can activate T-cells and regulatory B-cells, which have been defined as tumor-promoting effects (Wang et al., 2019). Thus, B-cell infiltration restrained tumor progression of LUAD, in accordance with the results of previous studies (J. Chen et al., 2020; Germain et al., 2014).

Macrophages, also named tumor-associated macrophages (TAMs), are the richest immune cell population of tumor tissues. M0 macrophages, the inactive TAMs, can polarize into inhibit-cancer-progression M1 macrophages or promote-cancer-progression M2 macrophages. M1 macrophages generate type I pro-inflammatory cytokines and possess anti-tumor functions. Meanwhile, M2 macrophages promote the matrix-remodeling through immunosuppression and thus favor tumor progression (Qian and Pollard 2010). In LUAD cells, M0 macrophages internalize tumor-derived exosomes and polarize into the M2 phenotype (Pritchard et al., 2020). Meanwhile, in LUAD tissues, M0 macrophages showed a significant infiltration in patients with poor prognosis (Liu et al., 2017; Mo et al., 2020). In our study, neither M1 nor M2 had a significant prognosis for LUAD patients. However, the correlation between poor prognosis and M0 macrophages was observed. In fact, M1 and M2 phenotypes present two extremes of a spectrum of functional states rather than certainly different cell types. Thus, our findings may reflect the polarizing function.

Dendritic cells are essential for the initiation and regulation of both innate and adaptive immune responses. As such, a number of approaches have been advanced to target dendritic cells to improve immunotherapy, such as antigens with immunomodulators that assemble and activate endogenous dendritic cells, as well as dendritic cell-based vaccines (Wculek et al., 2020). In LUAD patients, the lack of resting dendritic cells in tumor tissues is linked with worse anti-*PD*-(*L*)1 response, leading to a poor prognosis (Leader et al., 2021). Mast cells are also critical to tumor angiogenesis as well as metastases (Paolino et al., 2019). Mast cells are considered key regulators of the cancer stroma and coordinators of anti-tumor immunity and have been involved in tumor cell innate characteristics. Therefore, mast cells are an under-recognized but very promising target for cancer immunotherapy (Lichterman and Reddy 2021). In LUAD, high mast cell infiltration is considered an indicator of a good prognosis (Welsh et al., 2005; Carlini et al., 2010; Shikotra et al., 2016). These findings are consistent with our study that resting dendritic cells and mast cells were enriched in the low-TRIG-score group, indicating that they might benefit from immunotherapy.

In our study, the immune checkpoint gene expressions are also considered to differ between the two subtypes. Our study formed a model featuring six TRG (*CCL17*, *CD40LG*, *CIITA*, *GDF10*, *SCGB3A1*, and *STC1*) and identified that it could forecast OS in LUAD patients. Four of the six TRG (*CCL17*, *CD40LG*, *CIITA*, and *STC1*) are reported to be linked with immune checkpoints. Immune checkpoint blockades, such as sole and dual *CTLA-4* and *PD-1/PD-L1* blockades, have already represented a clinical benefit for several cancers including LUAD (Skoulidis et al., 2018; de Miguel and Calvo 2020). Chemokine (C–C motif) ligand 17 (*CCL17*), also named T(H)2-attracting chemokine (*TARC*), can recruit regulatory T-cells to TME. Regulatory T-cell accumulation in TME is reported to reduce anti-tumor immune response and is considered to be an essential driver of tumor immune evasion (Robles et al., 2020). Meanwhile, patients treated with combined immune checkpoint inhibitors represent the lowest expression of *CCL17* (Fiegle et al., 2019). In advanced melanoma patients treated with dendritic cell-based therapy, high serum levels of *CCL17* are related to improved progression-free survival (Cornforth et al., 2009). The *CD40* receptor and its ligand *CD40L*, widely expressed in various cells, is one of the master molecular pairs of the stimulatory immune checkpoint. The *CD40*/*CD40L*-targeted therapies show promising clinical efficacy in LUAD (Tang et al., 2021). The class II trans-activator (*CIITA*) is the most crucial regulator of the major histocompatibility complex (MHC) gene expression. In LUAD, loss of *CIITA* reduced cancer cell-specific MHCII and transformed LUAD from anti-*PD*-*1*-sensitive to anti-*PD*-*1*-resistant (Johnson et al., 2020). Expression of tumor stanniocalcin 1 (*STC1*) is reported to be related to immunotherapy efficacy and is negatively linked with patient survival in LUAD by tumor immune evasion and immunotherapy resistance. In murine tumor models, a gain of *STC1* favors tumor progression and allows tumor resistance to checkpoint blockade (Lin et al., 2021). All these studies correspond to our observations that high expression of *CCL17*, the *CD40* receptor-ligand gene (*CD40LG*), and *CIITA* and low expression of *STC1* are found in the low-TRIG-score group, indicating that patients in the low-TRIG-score group might benefit from immunotherapy. Another two TRIGs in this model, *SCGB3A1* (alias *HIN-1*) and *GDF10*, are both considered as tumor immune suppressors, which are correlated with clinicopathological variables (Garcia-Baquero et al., 2013; Cheng et al., 2016). These are consistent with our observations that high expressions of *SCGB3A1* and *GDF10* are identified in the low-TRIG-score group. Combined with our findings, these two TRIGs might have the potential to respond to immune checkpoint inhibitors.

Our study still had some limitations. First, all analyses were based on data from public databases. Thus, the results might have an innate case selection bias. Reliable *in vitro* and *in vivo* experiments along with large-scale prospective clinical trials are required to confirm our findings. Moreover, data on several critical clinical variables, including neoadjuvant chemotherapy, surgery, chemotherapy, targeted therapy, and immunotherapy, were unavailable in most datasets, which may have exerted an influence on the prognosis of immune responses.

## Data Availability

Publicly available datasets were analyzed in this study. These data can be found here: The Cancer Genome Atlas (TCGA) database, https://portal.gdc.cancer.gov/, the Gene Expression Omnibus (GEO) database, https://www.ncbi.nlm.nih.gov/geo/ (ID: GSE3141, GSE31210, GSE30219, GSE37745), and Search Tool for the Retrieval of Interacting Genes (STRING), version 11.0. https://string-db.org/the ImmPort Resource website. https://www.immport.org/shared/genelists.
