# Supplementary figures and images for: Identification of TRP-Related Subtypes, Development of a Prognostic Model, and Characterization of Tumor Microenvironment Infiltration in Lung Adenocarcinoma
Source: Front Mol Biosci. 2022 May 10;9:861380. doi: 10.3389/fmolb.2022.861380 (PMC9127446; doi:10.3389/fmolb.2022.861380)

Type 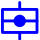 high 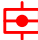 low

$p < 2.22\text{e-}16$

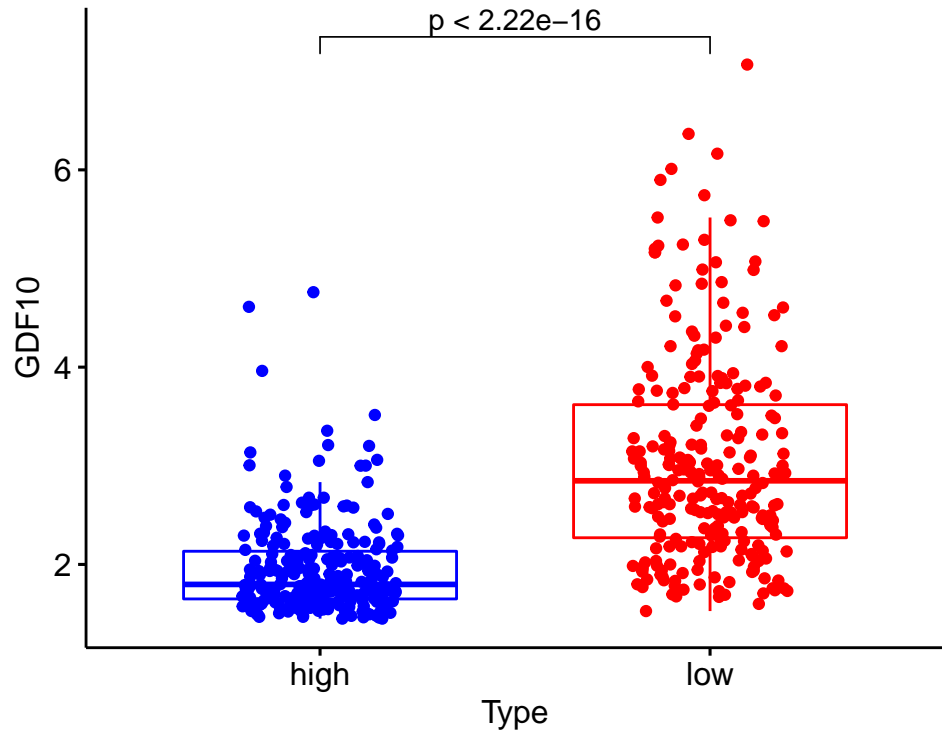

Supplement: Supplementary file 1 [file DataSheet2.PDF]

Type 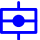 high 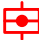 low

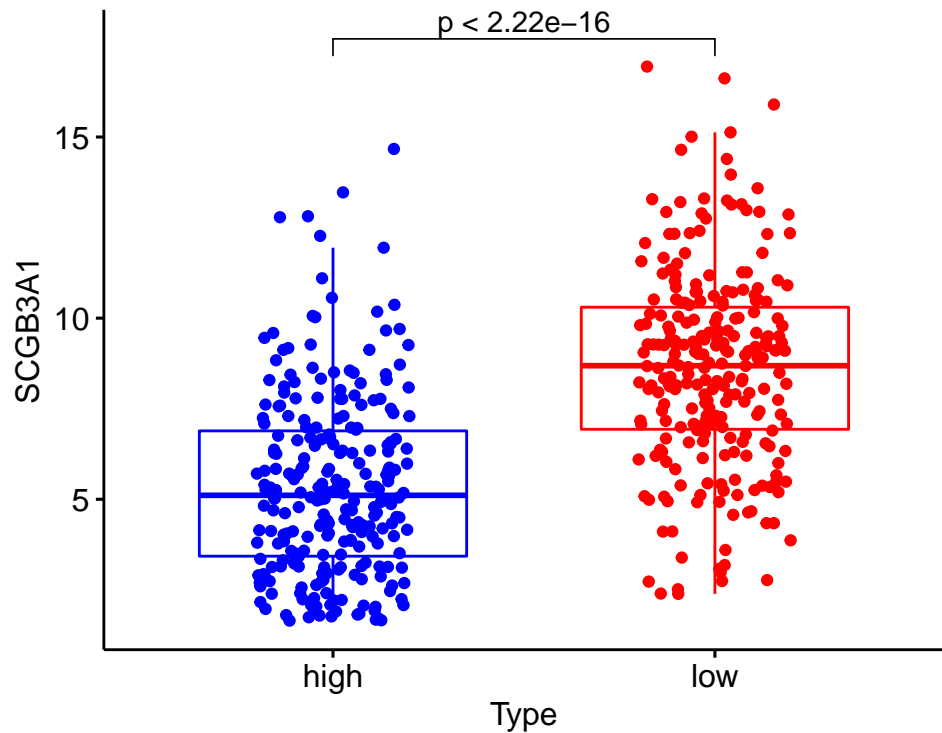

Supplement: Supplementary file 2 [file DataSheet4.PDF]

Altered in 211 (39.4%) of 535 samples.

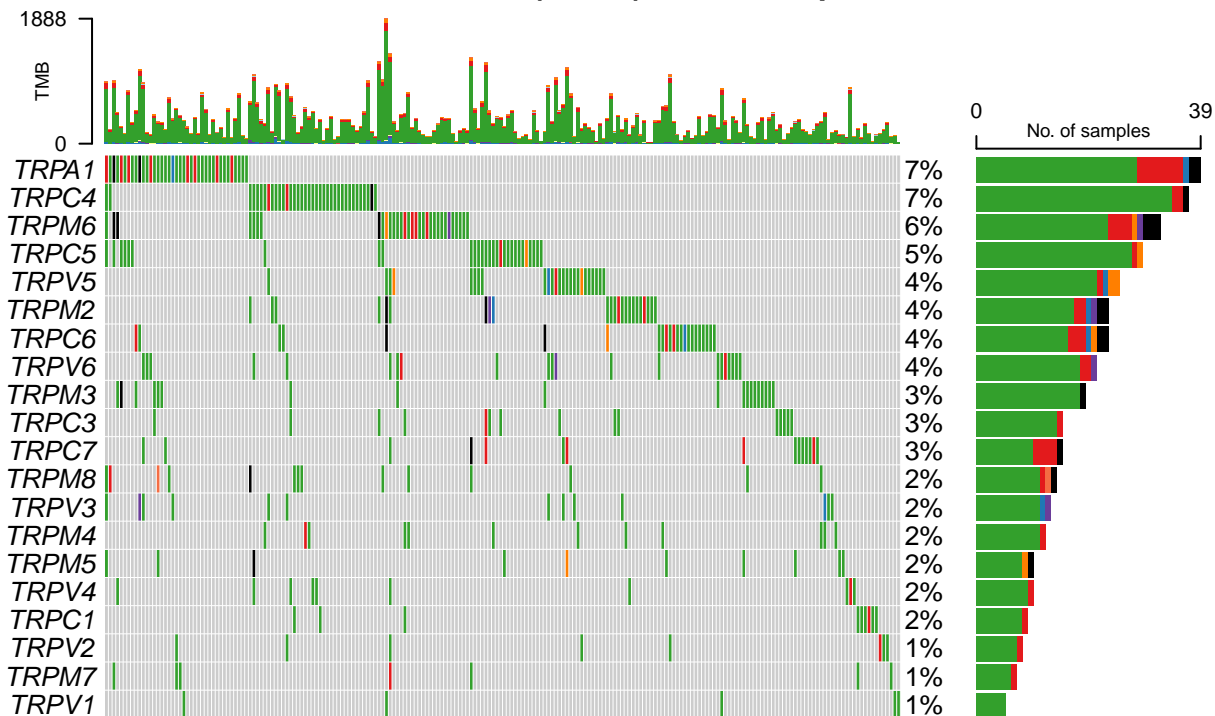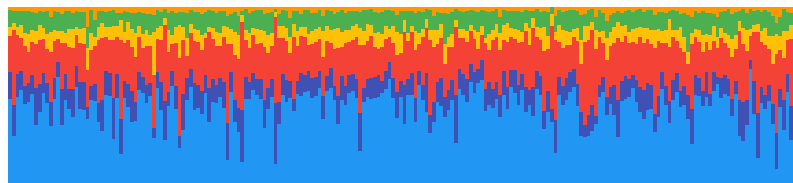

Supplement: Supplementary file 3 [file DataSheet3.PDF]

Type 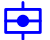 high 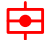 low

$p < 2.22\text{e-}16$

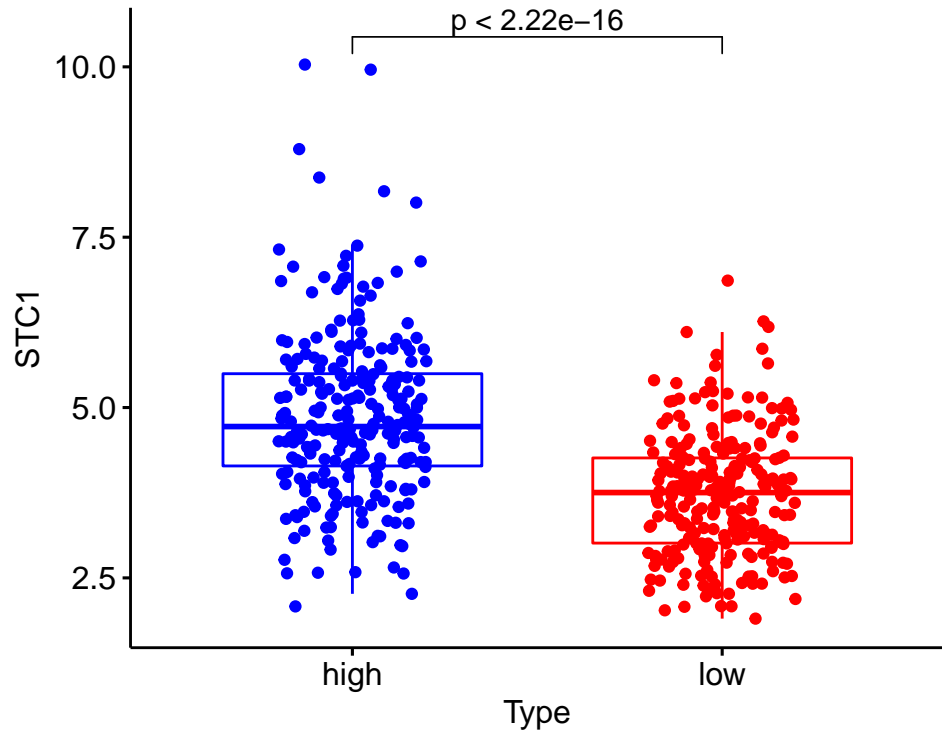

Supplement: Supplementary file 5 [file DataSheet5.PDF]
