# Supplementary material for: Identification of TRP-Related Subtypes, Development of a Prognostic Model, and Characterization of Tumor Microenvironment Infiltration in Lung Adenocarcinoma
Source: Front Mol Biosci. 2022 May 10;9:861380. doi: 10.3389/fmolb.2022.861380 (PMC9127446; doi:10.3389/fmolb.2022.861380)
Supplement: Supplementary file 4 [file DataSheet1.PDF]

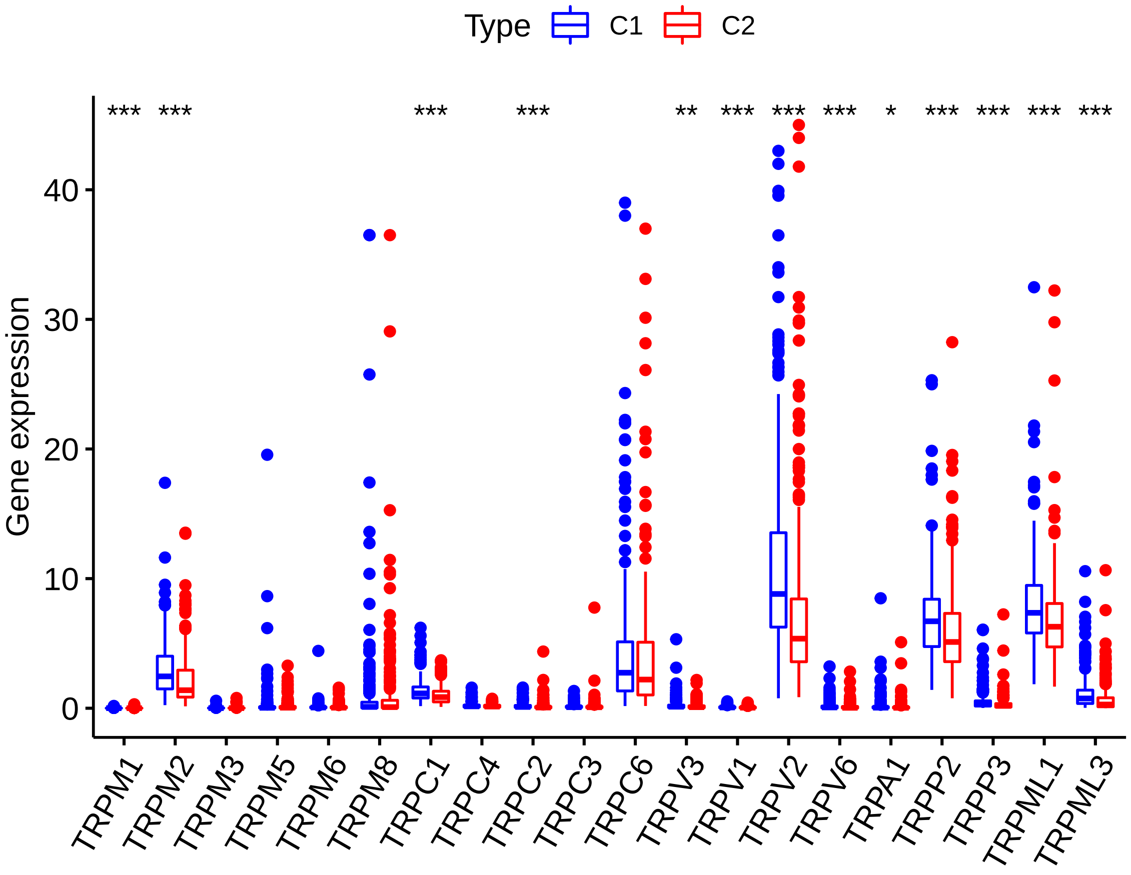

Type
C1
C2
Gene expression
0
10
20
30
40
***
***
***
***
**
***
***
***
*
***
***
***
***
TRPM1
TRPM2
TRPM3
TRPM5
TRPM6
TRPM8
TRPC1
TRPC4
TRPC2
TRPC3
TRPC6
TRPV3
TRPV1
TRPV2
TRPV6
TRPA1
TRPP2
TRPP3
TRPML1
TRPML3
